# Perceptions and reasons for quitting and transitioning between smoking and smokeless tobacco products: Findings from four waves of the ITC Bangladesh survey

**DOI:** 10.18332/tid/159137

**Published:** 2023-02-17

**Authors:** Daniel T. H. Chen, Nigar Nargis, Geoffrey T. Fong, Syed Mahfuzul Huq, Anne C. K. Quah, Filippos T. Filippidis

**Affiliations:** 1 Public Health Policy Evaluation Unit, School of Public Health, Imperial College London, London, United Kingdom; 2 Primary Care Epidemiology, Nuffield Department of Primary Care Health Sciences, University of Oxford, Oxford, United Kingdom; 3 American Cancer Society, Atlanta, United States; 4 Department of Psychology, University of Waterloo, Waterloo, Canada; 5 School of Public Health Sciences, University of Waterloo, Waterloo, Canada; 6 Ontario Institute for Cancer Research, Toronto, Canada; 7 Country Office of World Health Organization, Dhaka, Bangladesh

**Keywords:** smoking, smokeless tobacco, dual use, poly tobacco use, tobacco control

## Abstract

**INTRODUCTION:**

Transitions between different tobacco products are frequent among tobacco users in Bangladesh; however, the reasons leading to such transitions and why they quit are not well researched. The aim of the study is to examine perceptions and reasons reported by tobacco users in Bangladesh to transition to other products or quit.

**METHODS:**

Data from four waves (2009–2015) of the International Tobacco Control (ITC) Bangladesh Survey were used. Repeated data on perceptions and reasons for exclusive cigarette (n=520), bidi (n=130), and SLT users (n=308) to either start using other products or quit were analyzed with sampling weights. The percentages of responses across waves were used to calculate the pooled proportion data using a meta-analysis approach.

**RESULTS:**

Common reasonsig for respondents switching to other tobacco products were influence of friends/family (73.8–86.0%), and curiosity (44.4–71.3%). The perceived calming effect of smoking cigarettes and bidis (43.2–56.9%), and the impression that bidis were less harmful (52.3%) and taste better (71.2%) were major reasons for exclusive SLT users to switch products. Health concerns (16.5–62.7%) and disapproval from friends/family (29.8–56.4%) were generally the main reasons for quitting. For smoked tobacco users, doctor’s advice (41.6%), package warning labels (32.3%), and price (32.4%) seemed to be the major driving factors to quit.

**CONCLUSIONS:**

Results highlight that the reasons for switching between tobacco products and quitting include social factors (e.g. friends/family) and (mis) perceptions regarding the products. Tobacco control policy could emphasize cessation support, increased price and education campaigns as key policies to reduce overall tobacco use in Bangladesh. Data from four waves (2009–2015) of the International Tobacco Control (ITC) Bangladesh Survey were used. Repeated data on perceptions and reasons for exclusive cigarette (n=520), bidi (n=130), and SLT users (n=308) to either start using other products or quit were analyzed with sampling weights. The percentages of responses across waves were used to calculate the pooled proportion data using a meta-analysis approach.

## INTRODUCTION

Tobacco use, whether smoking or smokeless, is widely regarded as the leading cause of preventable morbidity and premature mortality worldwide, particularly in low- and middle-income countries (LMICs)^1,2^. The World Health Organization (WHO) estimated that in 2022 the South-East Asia Region accounted for more than 22% of global smokers aged ≥15 years and was home to 90% of the world’s smokeless tobacco (SLT) users^3^. In South-East Asia, tobacco is consumed in diverse forms, including smoked tobacco (ST), such as cigarettes, bidis (a cheaper hand-rolled substitute for cigarettes) and SLT, which covers a variety of tobacco containing products that are used orally or nasally without combustion^3,4^.

Although the proportion of tobacco users has declined in most South-East Asian countries in recent decades^1^, the growing tobacco market and the variety of products used within this region have led to concurrent use of multiple tobacco products and transitions of use between products^4-7^. A large number of tobacco users in South-East Asian countries were persistent users and unwilling to quit despite awareness of smoking hazards^8^. This is especially prominent in Bangladesh, one of the highest tobacco-consuming countries in the region, where there were 37.8 million (35.3%) adults consuming tobacco products (mainly cigarettes, bidis, and SLT)^9^ in 2017.

SLT is the most commonly used form of tobacco in Bangladesh, accounting for a substantial portion of overall tobacco use in the nation^3^. Available products include betel quid with tobacco, zarda, gul, sada pata, and khoinee, the majority of which are also available in neighboring India and Myanmar^10^. As with ST, SLT is found to be associated with adverse health effects such as the development of cancer, cardiovascular disease, hypertension, and adverse pregnancy outcomes^11,12^. However, almost one in ten (9.3%) tobacco users in Bangladesh is a dual user of both ST and SLT, and one in eleven (8.8%) is a poly-user of two or more tobacco products^6^. Furthermore, regardless of the country’s high health and economic burden from tobacco use, quit ratios have remained low, even though the population of former smokers has increased in most South-East Asian countries in recent years^13,14^.

Most studies, to date, have explored factors associated with different tobacco use patterns and quitting of smoked and smokeless tobacco^7,13,15^. Shared traits of these users in the South-East Asian context were that they were older, of lower education level, in poverty, and higher intentions to quit^13,15^. However, no studies have focused on users’ perceptions and reasons for changes in their tobacco use behavior. Within the context of Bangladesh, which represents a substantial proportion of the region’s tobacco market, it is essential to understand both person/product-level factors (i.e. product knowledge, appeal, and perceived harm, etc.) and contextual factors (i.e. policy restrictions, affordability and product availability, etc.) that influence the behavioral changes of these users for tobacco product substitution/initiation and nicotine seeking^16^. This will improve our understanding of the factors that help to reduce overall use and lead to quitting, and will inform policies on tobacco control to maximize their impact. Such information can also inform tobacco control policies in neighboring South-East Asian countries with similar tobacco use profiles.

In light of this, this study aimed to explore factors of product transitions, reasons and perceptions about quitting, using cohort data from adult smokers in Bangladesh.

## METHODS

### Data and sample

Data come from all four waves of The International Tobacco Control (ITC) Survey in Bangladesh. The ITC Bangladesh Survey uses a nationally representative probability sample of adult tobacco users and non-users aged ≥15 years, recruited using a multistage cluster sampling design since the first wave in 2009. Participants were re-contacted in 2010, 2011–2012 and 2014–2015, to complete follow-up questionnaires. The retention rates were fairly high, between 87.1% and 94.0%. Details on survey interview procedures, questionnaires, sampling, weighting, and information on accessing the data are available on the ITC website (https://itcproject.org/countries/bangladesh/) and the technical reports^17-20^.

To explore respondent-level changes in tobacco product use and reasons related to the transitions of use among Bangladeshi adults throughout the surveys, we used a balanced sample of respondents present in all four waves yielding a total of 958 respondents that were followed up since the first wave on perceptions and reasons for tobacco use transition to other smoked or smokeless products or to quitting. All tobacco use-related questions and data were retrieved from the tobacco users’ survey, except for data on SLT use in Waves 1 and 2, which were partially recorded and derived from the non-users’ surveys due to questionnaire design.

### Measures

*Sociodemographic characteristics*

Sociodemographic characteristics examined were sex, age group (15–17, 18–24, 25–39, 40–54, and ≥55 years), residence (urban, rural), marital status (married, single or living alone), and education level (illiterate, 1–8 years or ≥9 years), and the CASHPOR housing index as a proxy of socioeconomic status (SES) (low, intermediate, high). The CASHPOR index of housing conditions was originally constructed by the ITC researchers and used as the basis for stratification of the population by socioeconomic status^21^.

*Current tobacco use*

In the current study, respondents were considered as current tobacco users if they reported current use of cigarettes, bidis or smokeless (SLT) products on a daily, weekly, or less than weekly basis (e.g. monthly), in the surveys. For users of smoking products, the question was: ‘Do you currently smoke cigarettes/bidis?’. Those who answered ‘yes’ (Wave 1) or ‘daily/weekly/less than weekly’ (Waves 2, 3, and 4) were categorized as current users. For the identification of SLT users, the relevant question asked in Waves 1 and 2 was: ‘In the past 6 months, have you used any smokeless products?’ (yes/no); and a response to the statement ‘I generally use SLT at least weekly.’ (yes/no). Those who responded ‘yes’ to both, were classified as current SLT users. In Waves 3 and 4, the question was asked: ‘Do you currently use SLT?’, with response options ‘daily’, ‘weekly’, or ‘less than weekly’ indicating current use. However, those who responded ‘no’ to all of the above, were classified as non-users or quitters.

*Perceptions and reasons for quitting and transitions*

A set of questions around perceptions and reasons why exclusive cigarette, bidis, and SLT users in Wave 1 transitioned to other tobacco products (either exclusively or as dual/poly users of other products) in subsequent waves, were assessed. Current tobacco users were asked a series of questions about why they started smoking cigarettes (Waves 2 to 4), bidis (Waves 2 to 4), or using SLT (Waves 3 to 4). Potential answers to the questions include the influence of friends/family; people on media (cigarettes and bidis); curiosity; to occupy time; the calming effects (cigarettes and bidis); sign of sophistication (cigarettes); the packaging (bidis and SLT); to reduce stress (SLT); to help quit tobacco (SLT); the taste (bidis and SLT); and considered to be less harmful (bidis and SLT). Respondents were asked to respond ‘yes’, ‘no’, and ‘don’t know’ to the series of questions in the relevant waves. Reasons for quitting in the following waves were assessed by a number of questions asking cigarette (Waves 2 to 3) and bidi (Waves 2 to 4) smokers, or SLT (Waves 3 to 4) users, whether any of the following reasons led them to think about quitting: concerns for health; concerns for others; fewer places to smoke; workplace restrictions (SLT); set an example for children; doctor’s advice; price; warning labels; friends/family disapprove; and society disapproves (SLT). The responses to these questions were ‘yes’, ‘no’, and ‘don’t know’.

It is worth noting that some questions were asked only in certain waves. Therefore, data were analyzed only among the waves for which data were available. Supplementary file Table 1 shows a list of all the full questions asked above in relation to perceptions and reasons for transitions/quitting in the ITC Bangladesh Survey.

### Statistical analysis

Descriptive data of the sample characteristics are shown as weighted percentages and 95% confidence intervals (CI).

The responses to questions around reasons for transitions between products and quitting were analyzed using repeated data from Waves 2 to 4, with Wave 1 as the base wave, of the ITC Bangladesh Survey. To obtain the pooled percentage of response across all waves, the Stata command metaprop was used to perform meta-analyses of the proportion data^22^. This command offers proper techniques for pooling percentage data using the random-effects model and permits computation of accurate binomial and score test-based confidence intervals when the estimated proportion of the sub-group is between 0 and 1^22^. All analyses were weighted using the ITC sampling weights respective to the waves analyzed. Weighted estimates are presented to ensure that results are representative of the Bangladeshi population of tobacco users.

In the current study, only male adults were included for analyses for exclusive cigarette and exclusive bidi smokers, considering the inadequate size of the female smoker sample (the prevalence of cigarette and bidi smoking among female adults was less than 2% in all waves of the survey).

## RESULTS

### Sample characteristics of respondents who transitioned

The analytical sample consisted of 958 exclusive users (520 male exclusive cigarette smokers, 130 male exclusive bidi smokers, and 308 male and female exclusive SLT users) recruited at Wave 1 and who had transitioned to using other tobacco products (either exclusively or as dual or poly users with other products) or quit at Waves 2 to 4. The breakdown of the numbers of respondents who transitioned in subsequent waves is presented in Supplementary file Table 2.

As presented in Table 1, most exclusive users who transitioned to using other tobacco products were of younger age (≤54 years), living in rural areas, and married. However, an exceptionally large proportion (73.1%) of exclusive bidi smokers who quit were of older age (≥55 years). Within this cohort sample, 34.8–40.7% of respondents who quit were from better housing conditions (higher SES). Percentages of transitions to using other products were higher among those who were illiterate or received less education (1–8 years) for most exclusive users (≥76.4%). Additionally, most exclusive SLT users who transitioned to smoking cigarettes were male (74.6%), while those who transitioned to bidi use or to quitting, were mainly female (68.5% and 73.4%, respectively).

**Table 1:** Sample characteristics of exclusive tobacco users at Wave 1, who transitioned to other products or to quitting at Waves 2–4 of the International Tobacco Control (ITC) survey Bangladesh, 2009–2015 (N=958)

Characteristics	Exclusive cigarette smokers at Wave 1* (Males only)			Exclusive bidi smokers at Wave 1* (Males only)			Exclusive SLT users at Wave 1*		
	Transition to bidis (N=110)	Transition to SLT (N=190)	Transition to quitting (N=220)	Transition to cigarettes (N=61)	Transition to SLT (N=47)	Transition to quitting (N=22)	Transition to cigarettes (N=23)	Transition to bidis (N=22)	Transition to quitting (N=263)
**Gender**									
Male							74.6 (65.8–81.5)	31.5 (18.3–49.1)	27.6 (22.6–33.3)
Female							25.4 (18.5–34.2)	68.5 (60.9–91.7)	73.4 (66.7–77.4)
**Age** (years)									
15–24	16.6 (10.7–24.7)	11.7 (7.8–17.1)	20.7 (15.9–26.6)	3.7 (1.5–12.4)	2.5 (1.0–5.6)	5.2 (3.6–14.3)	7.9 (3.9–17.0)	2.1 (0.5–9.3)	10.8 (7.5–15.1)
25–39	28.6 (20.9–37.7)	48.7 (41.7–55.8)	43.2 (36.9–49.8)	21.7 (13.1–33.7)	10.3 (3.2–15.5)	6.0 (3.3–15.6)	17.5 (14.0–36.8)	3.1 (3.5–8.9)	33.9 (28.4–39.8)
40–54	30.4 (22.5–39.5)	19.5 (14.4–25.7)	18.1 (13.5–23.7)	41.8 (30.2–54.3)	49.4 (43.5–55.4)	15.8 (5.2–26.6)	64.3 (56.8–74.9)	6.8 (3.1–13.6)	33.3 (27.9–39.2)
≥55	24.5 (17.4–33.4)	20.2 (15.0–26.5)	18.0 (13.4–23.6)	32.9 (22.4–45.4)	37.8 (33.5–44.6)	73.1 (61.9–87.4)	10.3 (5.3–17.8)	88.0 (71.1–90.2)	22.0 (17.1–27.4)
**Residence**									
Urban	18.3 (12.1–26.7)	19.2 (14.2–25.4)	27.4 (21.9–33.6)	7.4 (2.7–17.2)	9.1 (2.4–14.0)	9.3 (1.4–13.2)	21.2 (16.0–30.3)	4.7 (2.7–12.5)	24.5 (19.7–30.1)
Rural	81.7 (73.3–87.9)	80.8 (74.6–85.8)	72.7 (66.4–78.1)	92.6 (82.8–97.3)	90.9 (86.0–97.6)	90.8 (90.8–98.6)	78.8 (59.7–84.0)	95.3 (88.3–99.3)	75.5 (69.9–80.3)
**Housing index**									
Low	26.6 (19.2–35.6)	31.9 (25.6–38.8)	25.3 (20.0–31.4)	47.1 (35.1–59.4)	44.4 (29.1–50.8)	29.4 (24.4–40.5)	32.9 (23.2–50.8)	5.2 (3.5–18.9)	29.5 (24.3–35.3)
Medium	35.5 (27.2–44.9)	38.7 (32.1–45.8)	34.1 (28.1–40.5)	37.2 (26.1–49.8)	41.3 (36.5–50.9)	35.8 (29.2–46.6)	1.2 (3.6–9.6)	74.6 (65.2–84.8)	32.6 (27.2–38.5)
High	37.8 (29.3–47.2)	29.4 (23.4–36.3)	40.7 (34.4–47.3)	15.7 (8.5–27.1)	14.3 (5.7–20.2)	34.8 (28.4–45.7)	66.0 (48.2–76.0)	20.2 (12.7–30.9)	37.9 (32.3–43.9)
**Education level** (years)									
Illiterate	22.1 (15.3–30.8)	20.2 (15.1–26.5)	18.8 (14.2–24.5)	57.7 (45.2–69.3)	49.0 (33.2–55.0)	43.0 (34.9–53.2)	26.0 (18.9–34.7)	68.6 (54.6–74.4)	41.7 (35.9–47.7)
1–8	60.7 (51.3–69.4)	61.0 (53.9–67.7)	45.7 (39.3–52.3)	33.6 (23.0–46.2)	49.6 (33.8–55.6)	51.0 (41.6–60.2)	50.5 (25.7–65.0)	21.1 (15.6–35.4)	49.3 (43.4–55.3)
≥9	17.2 (11.1–25.4)	18.8 (13.8–25.0)	35.5 (29.4–42.0)	8.7 (3.5–18.8)	1.4 (0.5–10.0)	6.0 (2.2–15.3)	23.6 (7.4–32.5)	10.3 (23.5–33.5)	9.0 (6.1–13.1)
**Marital status**									
Married	82.3 (74.0–88.4)	81.8 (75.6–86.6)	78.56 (72.6–83.5)	95.5 (86.3–99.2)	97.4 (83.7–99.1)	94.81 (85.69–99.52)	73.60 (64.88–90.87)	72.52 (60.87–84.61)	81.37 (80.92–94.50)
Single/live alone	17.7 (11.6–26.0)	18.2 (13.4–24.4)	21.5 (16.5–27.4)	4.5 (0.9–13.7)	2.6 (1.1–6.3)	5.19 (2.61–14.31)	26.40 (19.13–35.12)	27.48 (15.39–39.13)	18.63 (14.31–23.89)

* Weighted % with 95% CI. Blank: no data.

N=520 for exclusive cigarette smokers. N=130 for exclusive bidi smokers. N=308 for exclusive SLT smokers. Male respondents only for exclusive cigarette and bidi smokers. SLT: smokeless tobacco.

### Perceptions and reasons for quitting and transition

**Figure 1:**
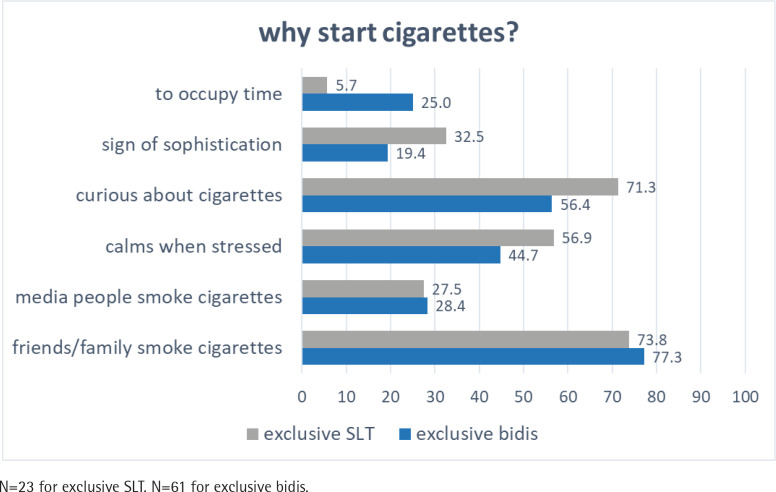
Perceptions and reasons for exclusive bidis and smokeless tobacco (SLT) users to start using cigarettes from Wave 2 (2009) to Wave 4 (2014) of the ITC-Bangladesh survey (pooled %)

**Figure 2:**
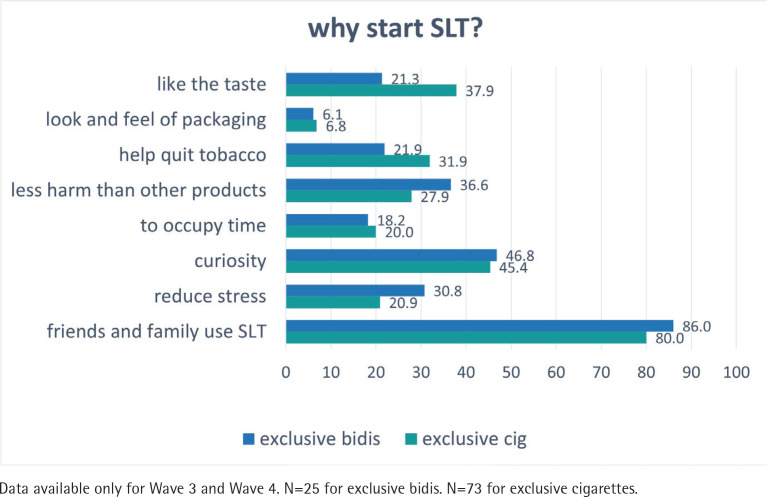
Perceptions and reasons for exclusive cigarettes and bidis users to start using smokeless tobacco (SLT) from Wave 3 (2011) to Wave 4 (2014) of the ITC-Bangladesh survey (pooled %)

**Figure 3:**
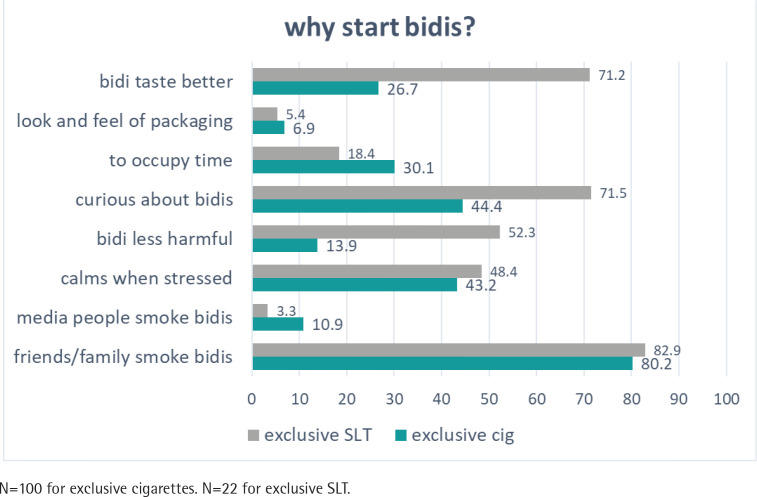
Perceptions and reasons for exclusive smokeless tobacco (SLT) and cigarette users to start using bidis from Wave 2 (2009) to Wave 4 (2014) of the ITC-Bangladesh survey (pooled %)

**Figure 4:**
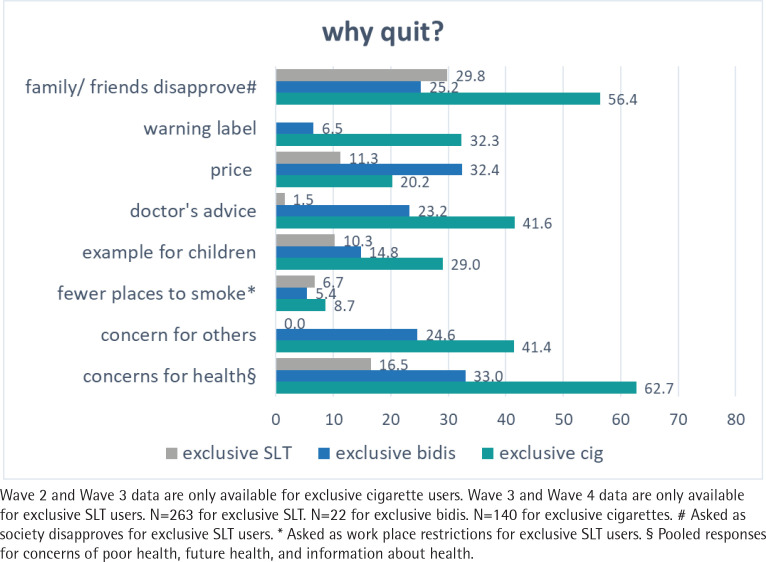
Perceptions and reasons for exclusive cigarettes, bidis, and smokeless tobacco (SLT) to quit from Wave 2 (2009) to Wave 4 (2014) of the ITC-Bangladesh survey (pooled %)

Figures 1 to 4 present the pooled proportions of perceptions and reasons for exclusive cigarette, bidis, and SLT users in Waves 2 to 4, to start using other products or to quit. Estimates with 95% CIs are presented in Supplementary file Table 3. As shown in Figure 1, the top three reasons for exclusive SLT users to start smoking cigarettes were the influence of friends/family (73.8%), curiosity (71.3%), and to calm stress (56.9%). The reasons for exclusive bidi smokers to start smoking cigarettes were generally the same, the proportions were 77.3%, 56.4%, and 44.7%, respectively, for the above reasons.

In Figure 2, the most common two reasons for exclusive bidi and exclusive cigarette smokers to start using SLT were friends/family (86.0% and 80.0%, respectively) and curiosity (46.8% and 45.4%, respectively). A larger proportion of exclusive cigarette smokers started SLT because of the better taste (37.9%), and because they thought that it helps quit tobacco use (31.9%); 36.6% exclusive bidi smokers started SLT because they thought it is less harmful, and 30.8% smokers considered that it reduces stress. Only around 6% of exclusive cigarette/bidi smokers started SLT because of the packaging warning characteristics, which is ranked in last place among all reasons.

In Figure 3, the top four reasons for exclusive SLT users to start smoking bidis were friends/family (82.9%), curiosity (71.5%), the impression that bidis taste better (71.2%), and thinking that bidis are less harmful (52.3%). Regarding exclusive cigarette users, the major reasons to start smoking bidis were friends/family (80.2%), curiosity (44.4%), calming of stress (43.2%), to occupy time (30.1%), and the impression that bidis taste better (26.7%).

Figure 4 describes the top reasons for exclusive users to quit. For exclusive cigarette smokers, the majority quit because of their concerns for health (62.7%), disapproval from friends/family (56.4%), followed by doctor’s advice and concerns for others (41.6% and 41.4%, respectively). The lowest ranked reason for exclusive cigarette smokers to quit was smoking bans (8.7%) in public places. As for exclusive bidi smokers, the leading reported reasons were price and health concerns (32.4% and 33.0%, respectively), followed by disapproval of friends/family, concern for others (25.2% and 24.6%, respectively), and doctor’s advice to quit (23.2%). Only around 5% of exclusive bidi smokers quit because of the warning labels and smoking bans. For exclusive SLT users, the majority quit because of their friends/family (29.8%), and concerns about health (16.5%). About 10% quit because of the price and to set an example for their children. Only 1.5% of exclusive SLT users quit because of the doctor’s advice, which was the least reported reason.

## DISCUSSION

Transitions between tobacco products are frequent among tobacco users in Bangladesh. Social factors were major reasons for quitting and transitioning. Curiosity and misperceptions of tobacco products were also drivers for users to switch between products. Furthermore, tobacco control measures such as price/tax, ban on Tobacco Advertisement Promotion & Sponsorship (TAPS) in entertainment media, and health education programs for awareness of the harmful effects, have an essential influence on the reported reasons for consumers to switch to, co-use or discontinue using different tobacco products.

Friends/family were the most common social factor for respondents switching to other tobacco products. This is in line with established evidence emphasizing that smoking behaviors are heavily impacted by peers and others close to the respondents’ social contacts, such as friends/family^23^. Such influence was found to be more relevant in developing countries and Asian cultures, where smoking is a social activity to develop social networks and a tool for networking^24^. However, social factors also act as a double-edged sword; tobacco users are prone to switching products due to peer influence and are also more inclined to quit because of their disapproval^23^. Our study results show that health concerns and disapproval from friends/family were also reported as important reasons for quitting. This may indicate that consumer education and anti-tobacco campaigns may be an effective policy to aid tobacco users to quit. A recent study identified education and raising public awareness around anti-tobacco advocacies in Bangladesh, would stand as a key opportunity against the culturally engrained acceptance of tobacco and SLT use^25^. Therefore, educational campaigns should focus on the social aspect of smoking behaviors.

Factors related with perceptions of harm, curiosity, and mistaken belief of the calming effect or stressreducing properties of tobacco products, were the major reasons for their transition between smoking and smokeless products. This might be driven by advertising strategies that promote the misleading impression that SLT or bidis are more appealing (taste better) than manufactured cigarettes^26^. Previous studies also pointed out that curiosity about tobacco products was highly associated with product initiation^27^. Receptivity to tobacco industry advertising and promotions may explain the high proportions of respondents susceptible to smoking due to curiosity^28^. This is an exceptionally a prominent reason why SLT users in Bangladesh start using smoked products. As noted in previous studies, this proportion increased with additional exposure to tobacco marketing throughout adolescence^29^. Educational campaigns and TAPS bans of bidis and smoking products, should be deemed critical policies and strengthened to discourage people from consuming tobacco products to reduce overall tobacco use prevalence in Bangladesh^30^.

Furthermore, results indicate that doctor’s advice accounted for a significant share of why bidi and cigarette smokers quit. Doctors’ and health professionals’ advice may be an effective strategy to combat misinformation/misconception and may have an impact on smoking behaviors, since many smokers identify health concerns as a primary reason for quitting. However, this was not the case with SLT users. Our results show that only 1.5% of SLT users received doctors’ advice to quit. Despite the high disease burden associated with SLT use in Bangladesh, access to doctors’ advice and cessation services are not yet widely available for SLT users in routine healthcare in Bangladesh^11,25^. To combat the high prevalence of SLT use and the frequent transition between smoking and smokeless products, authorities should place a greater emphasis on integrating SLT cessation services on treating nicotine dependence^25^ and provide support for high-risk populations (e.g. rural female SLT users or older male SLT users transitioning to cigarette use).

The status of tobacco control policies appears to be an important factor in influencing smoking behaviors and tobacco use. There is clear evidence that smoking restrictions in workplaces and public places contribute to smoking cessation and reduce tobacco use prevalence^1^. Large pictorial warnings with strong messages are also proven to effectively persuade smokers to quit smoking and increase compliance with smoke-free laws^31,32^. However, amid all responses, smoking restrictions and packaging warning labels were generally not a frequently cited reason for smokers to quit (particularly exclusive bidi smokers).

Bangladesh has implemented various policies and initiatives since the implementation of the Tobacco Control Act in 2005. A series of policy amendments have then been brought forward since the first ITC Bangladesh Survey in 2009, including amendments to the Act in 2013 (after Wave 3) and framing new guidelines in 2015 (before Wave 4), to supersede the original 2005 Act and bring the nation closer to compliance with the WHO Framework Convention on Tobacco Control (FCTC). It was not until 2013 that the list of smoke-free public areas was largely expanded and the requirement imposed that all tobacco products have warning labels that cover at least 50% of the total display space on the packaging^33^. Therefore, implementations of smoke-free laws and regulations on warning labels may have been less effective at the time when the surveys were conducted and should be continuously reinforced to further reduce tobacco consumption. However, up until now, restrictions on tobacco use in the transport area and public places, covers mainly smoked tobacco but not SLT^11,33^.

Generally, in many LMICs, smoke-free policies are often poorly implemented or enforced^34^. Social norms in most homes in LMICs still permit smoking indoors. Therefore, continuous reinforcements on expanding smoke-free policies to cover all public places and warning labels on alternative products, including bidis and SLT, should be enacted to curb the growing prevalence of use and to increase people’s awareness of the harms of tobacco in Bangladesh.

Regarding price, it was considered a driving factor for bidi smokers to quit but was not a frequently cited reason for cigarette and SLT users. Despite the increase in taxes and prices of tobacco products at the time of the surveys from 2009 to 2015 in Bangladesh^35^, cigarettes became more affordable as a result of the rising economy and income growth^4^. Cheaper brands and products are becoming increasingly popular, which may explain why price was not reflected in the respondents’ reasons for quitting. Taxation and price increases have been shown to be powerful motivators for reducing tobacco use and encouraging quitting^1^. Particularly in LMICs, such as Bangladesh, where taxation on tobacco products, especially on SLT, remains low^35^, increasing taxes and prices of all types of tobacco products is essential to ensure that the affordability of tobacco products continues to decline.

To our knowledge, this is the first cohort study exploring perceptions and reasons for transitions between smoked and smokeless tobacco products and quitting among adult smokers in Bangladesh. The current study examined a range of factors that were considered important by the respondents at the time of the study. However, the landscape of tobacco control policies in Bangladesh is changing. The perceptions and reasons do not necessarily reflect the actual effectiveness of various policies. Regardless, these findings can be informative of future tobacco control policies and could be incorporated into the revision of current regulations in the country to strengthen existing policies that appeal to the users’ concerns and motivations to quit. Furthermore, the factors influencing decisions about transitions and quitting may be shared among neighboring LMICs with similar tobacco use landscapes.

### Limitations

This study has some limitations. All results were meta-analyzed and pooled from all following waves after the initial survey, to maximize the representability of available data. Nevertheless, some questions were only asked of specific subsets of users; consequently, numbers may not be directly comparable between each user group. Due to this, response rankings were used to identify the most likely reasons when comparing across distinct exclusive user groups. Additionally, our analysis was based on self-reported data, which may be subject to information or recall bias. Perceptions and reasons for quitting and tobacco use could also change over time, given the fact that Bangladesh has made great progress between 2009 and 2015, when the respondents were assessed. Lastly, quitting was self-reported and defined as 30-day abstinence; we cannot verify that respondents remained abstinent from smoking.

## CONCLUSIONS

Influence of friends/family, curiosity, and misleading beliefs regarding tobacco products play important roles in the initiation and switching between products in Bangladesh. On the other hand, health concerns, friends/family disapproval, doctors’ advice, and package warning labels were seen to be effective reasons in encouraging cessation, especially for smoking products. Although Bangladesh has made notable progress in adopting various tobacco control policies during the past decades, the implementation of policies could be strengthened towards full compliance with FCTC. Understanding initiation to and transition between products could inform such policies, for example in terms of taxation levels across products, to reduce tobacco use in the country.

## Data Availability

In each country participating in the International Tobacco Control Policy Evaluation (ITC) Project, the data are jointly owned by the lead researcher(s) in that country and the ITC Project at the University of Waterloo. Data from the ITC Project are available to approved researchers, 2 years after the date of issuance of cleaned data sets by the ITC Data Management Centre. Researchers interested in using ITC data are required to apply for approval by submitting an International Tobacco Control Data Repository (ITCDR) request application and subsequently to sign an ITCDR Data Usage Agreement. The criteria for data usage approval and the contents of the Data Usage Agreement are described online (http://www.itcproject.org). The authors of this article obtained the data following this application process. They did not have any special access privileges. Others would be able to access the data in the same manner.
