# Supplementary material for: Perceptions and reasons for quitting and transitioning between smoking and smokeless tobacco products: Findings from four waves of the ITC Bangladesh survey
Source: Tob Induc Dis. 2023 Feb 17;21:25. doi: 10.18332/tid/159137 (PMC9936606; doi:10.18332/tid/159137)
Supplement: Supplementary file 1 [file TID-21-25-s1.pdf]

APPENDICES

Appendices to - Perceptions and reasons for quitting and transitioning between smoking and smokeless tobacco products: Findings from four waves of the ITC Bangladesh Survey

Appendix Table 1: Questions on starting/quitting tobacco use in the ITC Bangladesh Survey

**Why did you start smoking cigarettes?**	friends/family smoke cigs
	media people smoke cigs
	calms when stressed
	curious about cigs
	sign of sophistication
	to occupy time
**Why did you start smoking bidis?**	friends/family smoke bidis
	media people smoke bidis
	calms when stressed
	bidi less harmful
	curious about bidis
	to occupy time
	look and feel of packaging
	bidis taste better
**Why start using smokeless tobacco?**	friends and family use SLT
	reduce stress
	curiosity
	to occupy time
	less harm than other tobacco products
	help quit tobacco
	look and feel of packaging
	like the taste
**Why quit using cigarettes/bidis/SLT? (Have any of the following reasons led you to think about quitting cigarettes/bidis/SLT)**	concerns for health†
	concern for others
	fewer places to smoke*
	example for children
	doctor's advice
	price
	warning label
	family/ friends disapprove#

Note: † pooled responses for concerns of poor health, concerns of future health, and information about health; * asked as workplace restrictions for exclusive SLT users; # asked as society disapproves for exclusive SLT users

Appendix Table 2: Numbers of respondents transitioned in different waves (male respondents only for exclusive cigarette and bidi smokers)

	Transition to cigarette use	Transition to bidi use	Transition to SLT use	Quitting
**Exclusive cigarette use in W1 (n=520)**				
N of transitions in Wave 2	—	52	117	80
N of transitions in Wave 3	—	42	41	83
N of transitions in Wave 4	—	16	32	57
**Exclusive bidi use in W1 (n=130)**				
N of transitions in Wave 2	30	—	22	8
N of transitions in Wave 3	21	—	19	5
N of transitions in Wave 4	10	—	6	9
**Exclusive SLT use in W1 (n=308)**				
N of transitions in Wave 2	6	14	—	171
N of transitions in Wave 3	7	4	—	64
N of transitions in Wave 4	10	4	—	28

Note: — no data points

Appendix Table 3: Perceptions and reasons for exclusive cigarette, bidis, and SLT users to start using other products or quit from Wave 2 (2009) to Wave 4(2014) of the ITC-Bangladesh survey (pooled % with 95% Confidence Intervals)

	Weighted Percentages (%) with 95% CI		
	**Exclusive cigarette smokers**	**Exclusive bidi smokers**	**Exclusive SLT users**
**Why did you start smoking cigarettes?**			
friends/family smoke cigs	—	77.3 (67.0 - 86.2)	73.8 (36.2 - 99.2)
media people smoke cigs	—	28.4 (18.0 – 40.0)	27.5 (9.3 - 49.7)
calms when stressed	—	44.7 (34.8 - 54.9)	56.9 (34.8 - 77.9)
curious about cigs	—	56.4 (40.4 - 71.8)	71.3 (23.1 - 100)
sign of sophistication	—	19.4 (9.4 - 31.6)	32.5 (2.0 - 73.3)
to occupy time	—	25.0 (15.4 - 35.9)	5.7 (1.8 - 16.2)
**Why did you start smoking bidis?**			
friends/family smoke bidis	80.2 (61.3 - 94.1)	—	82.9 (55.6 - 99.7)
media people smoke bidis	10.9 (6.6 – 16.0)	—	3.3 (0.5 - 17.5)
calms when stressed	43.2 (27.9 - 59.2)	—	48.4 (20.2 – 80.3)
bidi less harmful	13.9 (8.6 – 20.0)	—	52.3 (15.0 - 88.5)
curious about bidis	44.4 (35.0 – 54.0)	—	71.5 (39.4 - 96.2)
to occupy time	30.1 (21.9 - 38.9)	—	18.4 (10.5 - 25.4)
look and feel of packaging	6.9 (3.2 - 11.6)	—	5.4 (0.6 - 9.9)
bidis taste better	26.7 (19.8 - 34.2)	—	71.2 (62.0 – 79.0)
**Why start using smokeless tobacco?**			
friends and family use SLT	80.0 (73.7 - 85.7)	86.0 (71 - 96.7)	—
reduce stress	20.9 (15.1 - 27.3)	30.8 (15.4 - 48.4)	—
curiosity	45.4 (38.0 - 52.8)	46.8 (29.3 - 64.7)	—
to occupy time	20.0 (14.3 - 26.3)	18.2 (6.0 - 24.2)	—
less harm than other tobacco products	27.9 (21.4 - 34.8)	36.6 (20.2 - 54.6)	—
help quit tobacco	31.9 (25.1 - 39.1)	21.9 (8.6 - 38.5)	—
look and feel of packaging	6.8 (3.4 - 11.2)	6.1 (2.1 - 18.4)	—
like the taste	37.9 (30.7 - 45.2)	21.3 (8.2 - 37.9)	—
**Why quit using cigarettes/bidis/SLT?**			
concerns for health†	62.7 (44.4 - 79.4)	33.0 (13.6 - 55.2)	16.5 (0.8 - 33.8)
concern for others	41.4 (34.3 - 48.7)	24.6 (5.0 - 35.4)	—
fewer places to smoke*	8.7 (4.9 - 13.3)	5.4 (0.5 – 21.0)	6.7 (1.2 - 15.3)
example for children	29.0 (22.6 - 35.9)	14.8 (2.5 - 32.2)	10.3 (3.3 - 19.9)
doctor's advice	41.6 (34.4 - 48.9)	23.2 (8.0 - 32.2)	1.5 (0.4 - 4.5)
price	20.2 (14.6 - 26.4)	32.4 (25.7 - 39.3)	11.3 (3.9 - 21.2)
warning label	32.3 (25.7 - 39.3)	6.5 (0.6 - 11.7)	—
family/ friends disapprove#	56.4 (49.1 - 63.6)	25.2 (9.4 - 34.4)	29.8 (18.4 - 42.5)

Note: † pooled responses for concerns of poor health, concerns of future health, and information about health; * asked as workplace restrictions for exclusive SLT users; # asked as society disapproves for exclusive SLT users; — no data points

© 2023 Chen D.T.H. et al.
